# Synthesis and crystal structure of (1,8-naphth­yridine-κ^2^ *N*,*N*′)[2-(1*H*-pyrazol-1-yl)phenyl-κ^2^ *N* ^2^,*C* ^1^]iridium(III) hexa­fluorido­phosphate di­chloro­methane monosolvate

**DOI:** 10.1107/S2056989019016773

**Published:** 2020-01-01

**Authors:** Yunfeng Ye, Guodong Tang, Jun Qian

**Affiliations:** a Jiangsu Nursing Vocational College, Huaian 223300, Jiangsu Province, People’s Republic of China; b Jiangsu Key Laboratory for Chemistry of Low-Dimensional Materials, Huaiyin, Normal University, Huaian 223300, Jiangsu Province, People’s Republic of China; c School of Chemistry and Chemical Engineering, Jiangsu University, Zhenjiang, 212013, Jiangsu Province, People’s Republic of China

**Keywords:** crystal structure, cyclo­metalated iridium complex, 1-phenyl­pyrazole, C—H⋯F hydrogen bonds

## Abstract

The solvated title salt, [Ir(C_9_H_7_N_2_)_2_(C_8_H_6_N_2_)]PF_6_·CH_2_Cl_2_, was obtained from the reaction between 1,8-naphthyridine (NAP) and an orthometalated iridium(III) precursor containing a 1-phenyl­pyrazole (ppz) ligand. The asymmetric unit comprises one [Ir(ppz)_2_(NAP)]^+^ cation, one PF_6_^−^ counter-ion and one CH_2_Cl_2_ solvent mol­ecule. The central Ir^III^ atom of the [Ir(ppz)_2_(NAP)]^+^ cation is distorted-octa­hedrally coordinated by four N atoms and two C atoms, whereby two N atoms stem from the NAP ligand while the ppz ligands ligate through one N and one C atom each. In the crystal, the [Ir(ppz)_2_(NAP)]^+^ cations and PF_6_^−^ counter-ions are connected with each other through weak inter­molecular C—H⋯F hydrogen bonds. Together with an additional C—H⋯F inter­action involving the solvent mol­ecule, a three-dimensional network structure is formed.

## Abstract 2

The coordination environment of the Ir^III^ atom in the complex cation is pseudo-octa­hedral, with an N_4_C_2_ coordination set.

## Chemical context   

Over the past two decades, transition-metal complexes have attracted considerable attention in both academia and industry (Dixon *et al.*, 2000 ▸). For example, *d* ^6^ iridium complexes with pseudo-octa­hedral coordination environments have been widely used in electroluminescent devices (sensors and light-emitting instruments) or photocatalysis because of their long excited-state lifetime, high quantum efficiency, luminescent colour adjustment and thermal stability (Lee *et al.*, 2013 ▸; Fan *et al.*, 2013 ▸). Among various iridium complexes, cyclo­metalated iridium(III) complexes are particularly attractive for the wide-range tunability of electronic structures *via* the rational mol­ecular design of different components (Zhu *et al.*, 2016 ▸). According to the set-up of cyclo­metalated iridium(III) cations with general formula [(N
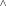
N)Ir(C
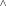
N)_2_]^+^ in which N
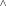
N refers to a di­imine ligand and C
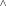
N refers to a cyclo­metalated ligand, the combination and variation of N
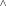
N and C
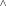
N ligands provides the opportunity to modulate the properties of the target complexes (Goswami *et al.*, 2014 ▸; Radwan *et al.*, 2015 ▸).

In our laboratory, a key motivation for studies in this area arises from our inter­est in cyclo­metalated iridium(III) complexes, which exhibit a strong conjugated system with a high degree of delocalized *π*-electrons. Thus, one can enhance the non-linear optical properties of a system through the inter­action between the *d* orbitals of Ir^III^ and the *π*-orbitals of an organic conjugated system (Liu *et al.*, 2018 ▸). Here we report the crystal structure of a solvated cyclo­metalated iridium(III) complex, [Ir(C_9_H_7_N_2_)_2_(C_8_H_6_N_2_)](PF_6_)·CH_2_Cl_2_, obtained from the reaction between an orthometalated iridium precursor ({(ppz)_2_Ir(*μ*-Cl)}_2_) (ppz = 1-phenyl­pyrazole) and 1,8-naphthyridine (NAP) as an auxiliary ligand.
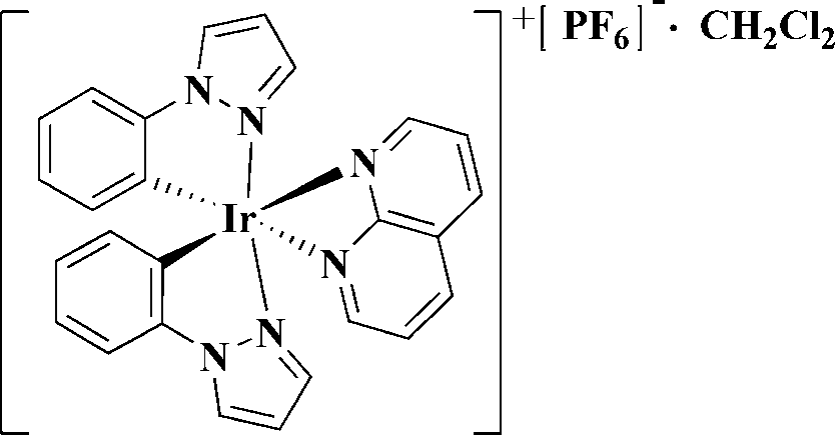


## Structural commentary   

**Figure 1:**
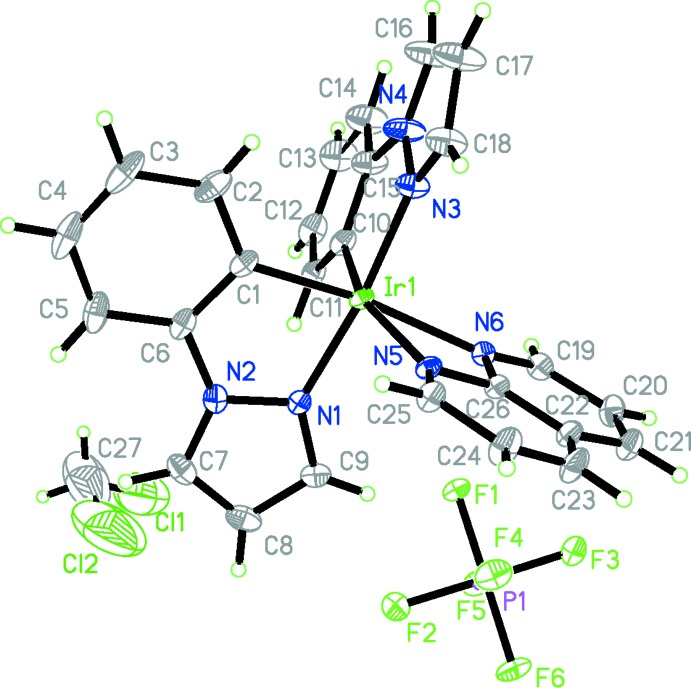
The structures of the mol­ecular entities in the title compound, with atom labelling. Displacement ellipsoids are drawn at the 30% probability level. H atoms are represented by spheres of arbitrary radius.

The asymmetric unit of the title cyclo­metalated iridium(III) complex is composed of one [Ir(ppz)_2_(NAP)]^+^ cation, one PF_6_^−^ counter-ion and one CH_2_Cl_2_ solvent mol­ecule. As shown in Fig. 1 ▸, the Ir^III^ atom is coordinated by four N and two C atoms in the form of a pseudo-octa­hedral [IrN_4_C_2_] polyhedron. The axial positions are occupied by two N atoms from two ppz ligands, while the equatorial plane is defined by two N atoms from the NAP ligand and two C atoms from the ppz ligands.

The bond lengths and angles related to the ppz ligand are normal and agree with the values in other cyclo­metalated iridium(III) compounds based on this ligand (see *Database survey* for details).

The average Ir—N_C
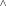
N_ (C
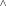
N refers to the ppz ligand) and Ir—C bond lengths are 2.013 and 2.008 Å, respectively, while the average Ir—N_N
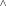
N_ (N
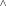
N refers to the NAP ligand) bond length is much longer at 2.208 Å. The bond angles around the Ir^III^ atom involving *cis*-arranged ligand atoms deviate clearly from 90° and range from 60.74 (10)° (the bite angle of the NAP ligand) to 110.71 (12)°, except for N1—Ir1—N5 with a value of 90.63 (11)°. Likewise, the bond angles N3—Ir1—N1, C1—Ir1—N6 and C10—Ir1—N5 of *trans*-oriented atoms are 173.28 (13), 170.06 (13) and 161.07 (13)°, respectively, and indicate a distortion from the ideal octa­hedral arrangement. The planes of the two planar ppz ligands (C1–C6/C7–C9/N1/N2, r.m.s. deviation of 0.0097 Å; C10–C15/C16–C18/N3/N4, r.m.s. deviation of 0.0562 Å) and the NAP ligand (r.m.s. deviation 0.389 Å) are 76.26 (8) and 70.63 (9)°, respectively, and thus deviate significantly from a perpendicular arrangement.

## Supra­molecular features   

**Figure 2:**
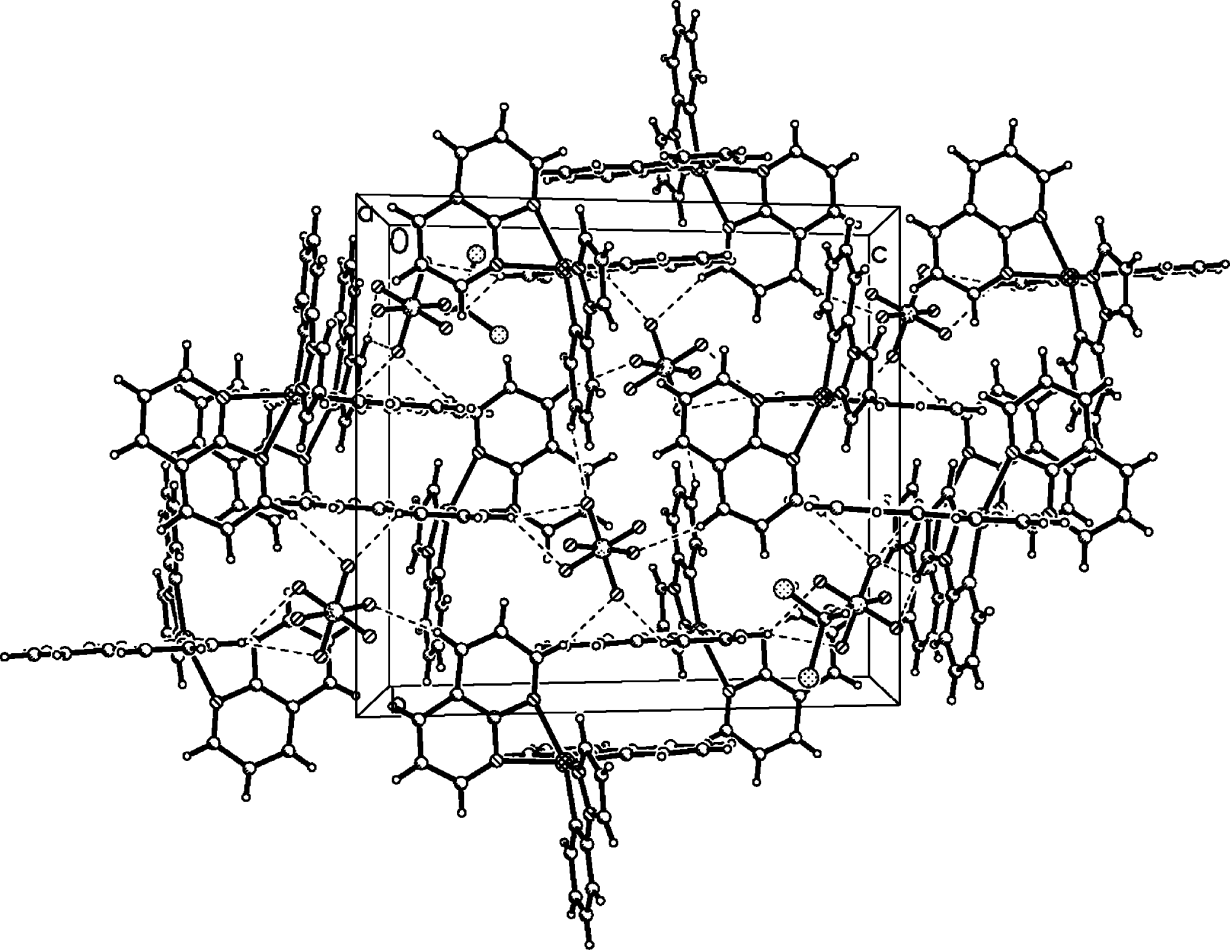
A packing diagram of the title compound in a view along the *a* axis, showing the three-dimensional supra­molecular network structure. C—H⋯F hydrogen bonds are shown as dashed lines.

In the crystal, the [Ir(ppz)_2_(NAP)]^+^ cations and PF_6_^−^ counter-ions are linked by six charge-assisted and partly bifurcated C—H⋯F hydrogen bonds (C16—H16*A*⋯F5^i^, C16—H16*A*⋯F6^i^, C9—H9*A*⋯F1, C9—H9*A*⋯F4, C7—H7*A*⋯F5^ii^, C25—H25*A*⋯F5^iii^; Table 1 ▸) into a three-dimensional supra­molecular network, as shown in Fig. 2 ▸. In addition, a similar hydrogen bond between the CH_2_Cl_2_ solvent mol­ecule and the PF_6_^−^counter-ion (C27—H27*A*⋯F2^iv^) consolidates this arrangement.

## Database survey   

A search of the Cambridge Structural Database (CSD, Version 5.39, updated November 2017; Groom *et al.*, 2016 ▸) for complexes containing an iridium(III) atom together with 1-phenyl­pyrazole ligand fragments yielded 36 hits. Among these, eight crystallize in the monoclinic system like the title compound. Five of them have similar chelating *N*,*N′*-ligands, *viz*. XAHXIP (Jiang *et al.*, 2010 ▸), KISYOC/KISZIX (Davies *et al.*, 2014 ▸), ROFZET (Sauvageot *et al.*, 2014 ▸) and JUPTIZ (Howarth *et al.*, 2015 ▸). Two compounds contain the same tetra­dentate ligand, *N*,*N′*-bis­(3,5-bis­(tri­fluoro­meth­yl)benzo­yl)hydrazide, and are *meso* and *rac* diastereomers, *viz*. NASQEG and NASQIK (Congrave *et al.*, 2017 ▸), and one compound is constructed solely by the 1-phenyl­pyrazole ligand, *viz*. OHUZAS (Tamayo *et al.*, 2003 ▸).

## Synthesis and crystallization   

The iridium dichloride bridge compound, [(ppz)_2_Ir(*μ*-Cl)]_2_, was synthesized following a reported literature procedure (Kwon *et al.*, 2005 ▸) by heating IrCl_3_·3H_2_O (1 equiv.) and 1-phenyl­pyrazole (2.3 equiv.) in a mixed solution of 2-eth­oxy­ethanol and water (*v*/*v* = 3/1) at 408 K.

1,8-Naphthyridine was synthesized by a slight modification of a reported procedure (Majewicz & Caluwe, 1975 ▸). The reaction of 1,3-cyclo­hexa­nedione and an excess of 2-amino­nicotinaldehyde in refluxing ethanol, which contains a few drops of methano­lic KOH, resulted in the 1,8-naphthyridine ligand.

The cyclo­metalated iridium(III) title complex (I) was synthesized from the reaction of [(ppz)_2_Ir(*μ*-Cl)]_2_ with 1,8-naphthyridine in a mixed solution of di­chloro­methane (CH_2_Cl_2_) and methanol (MeOH) (*v*/*v* = 2/1) at 358 K with KPF_6_ as counter-ion through metathesis. The reaction process was monitored by thin layer chromatography. After the reaction was complete, the mixture was dried under vacuum and separated by column chromatography on silica gel with CH_2_Cl_2_/petroleum ether (*v*/*v* = 4/1) as eluent. The pure product of the cyclo­metalated iridium(III) complex was obtained as a dark-yellow solid. Single crystals were grown by inter-diffusion between *n*-hexane and a di­chloro­methane solution of the pure solid with CH_2_Cl_2_/hexane (*v*/*v* = 1/1) as buffer solution at room temperature. Compared to the direct benign/inert solvents reaction system, here the inter-diffusion method was applied as a mild way for the crystallization of the title complex. The use of the buffer solution ensures stable conditions for the crystallization of co-responsive constituents (Nie *et al.*, 2019 ▸). Therefore, well-shaped crystals of complex(I) can be obtained from the buffer area.

Elemental analysis for C_27_H_22_Cl_2_F_6_IrN_6_P (found): C, 36.86; H, 2.63; N, 10.19%; (calculated): C, 37.65; H, 2.62; N, 10.12%.

## Refinement   

Crystal data, data collection and structure refinement details are summarized in Table 2 ▸. Carbon-bound H-atoms were placed in calculated positions (C—H = 0.93 Å for [Ir(ppz)_2_(NAP)]^+^ cation, C—H = 0.97 Å for CH_2_Cl_2_ solvent mol­ecule) and were included in the refinement in the riding-model approximation, with *U*_iso_(H) set to 1.2*U*_eq_(C).

## Supplementary Material

Crystal structure: contains datablock(s) I. DOI: 10.1107/S2056989019016773/wm5533sup1.cif

Structure factors: contains datablock(s) I. DOI: 10.1107/S2056989019016773/wm5533Isup3.hkl

CCDC reference: 1874317

Additional supporting information:  crystallographic information; 3D view; checkCIF report

## Figures and Tables

**Table 1:** Hydrogen-bond geometry (Å, °)

*D*—H⋯*A*	*D*—H	H⋯*A*	*D*⋯*A*	*D*—H⋯*A*
C9—H9*A*⋯F1	0.93	2.47	3.239 (4)	140
C9—H9*A*⋯F4	0.93	2.48	3.386 (5)	164
C16—H16*A*⋯F5^i^	0.93	2.46	3.018 (5)	118
C16—H16*A*⋯F6^i^	0.93	2.51	3.418 (6)	167
C7—H7*A*⋯F5^ii^	0.93	2.46	3.201 (5)	136
C25—H25*A*⋯F5^iii^	0.93	2.32	3.215 (4)	160
C27—H27*A*⋯F2^iv^	0.97	2.52	3.370 (13)	146

Symmetry codes: (i) 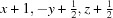; (ii) 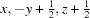; (iii) 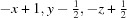; (iv) 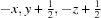.

**Table 2:** Experimental details

Crystal data	
Chemical formula	[Ir(C_9_H_7_N_2_)_2_(C_8_H_6_N_2_)]PF_6_·CH_2_Cl_2_
*M*_r_	838.57
Crystal system, space group	Monoclinic, *P*2_1_/*c*
Temperature (K)	293
*a*, *b*, *c* (Å)	12.1222 (3), 15.5510 (4), 17.1579 (5)
β (°)	105.313 (1)
*V* (Å^3^)	3119.64 (14)
*Z*	4
Radiation type	Mo *K*α
μ (mm^−1^)	4.57
Crystal size (mm)	0.20 × 0.18 × 0.15

Data collection	
Diffractometer	APEXII CCD area detector
Absorption correction	Multi-scan (*SADABS*; Bruker, 2016 ▸)
*T*_min_, *T*_max_	0.417, 0.504
No. of measured, independent and observed [*I* > 2σ(*I*)] reflections	36216, 6387, 5528
*R*_int_	0.032
(sin θ/λ)_max_ (Å^−1^)	0.626

Refinement	
*R*[*F* ^2^ > 2σ(*F* ^2^)], *wR*(*F* ^2^), *S*	0.024, 0.062, 1.04
No. of reflections	6387
No. of parameters	388
H-atom treatment	H-atom parameters constrained
Δρ_max_, Δρ_min_ (e Å^−3^)	1.29, −1.04

Computer programs: *APEX3* and *SAINT* (Bruker, 2016 ▸), *SHELXT* (Sheldrick, 2015*a* ▸), *SHELXL* (Sheldrick, 2015*b* ▸), *PLATON* (Spek, 2009 ▸) and *publCIF* (Westrip, 2010 ▸).

## supplementary crystallographic information

### Crystal data

**Table:**

[Ir(C_9_H_7_N_2_)_2_(C_8_H_6_N_2_)]PF_6_·CH_2_Cl_2_	*F*(000) = 1624
*M**_r_* = 838.57	*D*_x_ = 1.785 Mg m^−^^3^
Monoclinic, *P*2_1_/*c*	Mo *K*α radiation, λ = 0.71073 Å
*a* = 12.1222 (3) Å	Cell parameters from 9905 reflections
*b* = 15.5510 (4) Å	θ = 2.9–26.4°
*c* = 17.1579 (5) Å	µ = 4.57 mm^−^^1^
β = 105.313 (1)°	*T* = 293 K
*V* = 3119.64 (14) Å^3^	Block, red
*Z* = 4	0.20 × 0.18 × 0.15 mm

### Data collection

**Table:**

APEXII CCD area detector diffractometer	5528 reflections with *I* > 2σ(*I*)
phi and ω scans	*R*_int_ = 0.032
Absorption correction: multi-scan (*SADABS*; Bruker, 2016)	θ_max_ = 26.4°, θ_min_ = 2.9°
*T*_min_ = 0.417, *T*_max_ = 0.504	*h* = −15→15
36216 measured reflections	*k* = −19→19
6387 independent reflections	*l* = −21→21

### Refinement

**Table:**

Refinement on *F*^2^	0 restraints
Least-squares matrix: full	Hydrogen site location: inferred from neighbouring sites
*R*[*F*^2^ > 2σ(*F*^2^)] = 0.024	H-atom parameters constrained
*wR*(*F*^2^) = 0.062	*w* = 1/[σ^2^(*F*_o_^2^) + (0.0254*P*)^2^ + 8.5285*P*] where *P* = (*F*_o_^2^ + 2*F*_c_^2^)/3
*S* = 1.04	(Δ/σ)_max_ = 0.002
6387 reflections	Δρ_max_ = 1.29 e Å^−^^3^
388 parameters	Δρ_min_ = −1.03 e Å^−^^3^

### Special details

**Table:**

Geometry. All esds (except the esd in the dihedral angle between two l.s. planes) are estimated using the full covariance matrix. The cell esds are taken into account individually in the estimation of esds in distances, angles and torsion angles; correlations between esds in cell parameters are only used when they are defined by crystal symmetry. An approximate (isotropic) treatment of cell esds is used for estimating esds involving l.s. planes.

### Fractional atomic coordinates and isotropic or equivalent isotropic displacement parameters (Å^2^)

**Table:**

	*x*	*y*	*z*	*U*_iso_*/*U*_eq_	
Ir1	0.69497 (2)	0.12122 (2)	0.36803 (2)	0.02071 (5)	
N1	0.5244 (3)	0.11978 (17)	0.35308 (18)	0.0227 (6)	
N2	0.4908 (3)	0.11594 (18)	0.42268 (19)	0.0279 (7)	
N3	0.8666 (3)	0.12796 (19)	0.3960 (2)	0.0324 (7)	
N4	0.9109 (3)	0.2077 (2)	0.4177 (3)	0.0446 (10)	
N5	0.6867 (3)	−0.00707 (18)	0.30844 (17)	0.0231 (6)	
N6	0.6822 (3)	0.11475 (18)	0.23884 (18)	0.0229 (6)	
C1	0.6907 (3)	0.1083 (2)	0.4841 (2)	0.0272 (8)	
C2	0.7798 (4)	0.1025 (3)	0.5545 (3)	0.0423 (11)	
H2A	0.8550	0.1019	0.5508	0.051*	
C3	0.7581 (6)	0.0977 (3)	0.6303 (3)	0.0552 (15)	
H3A	0.8190	0.0936	0.6763	0.066*	
C4	0.6483 (6)	0.0990 (3)	0.6380 (3)	0.0541 (15)	
H4A	0.6355	0.0960	0.6891	0.065*	
C5	0.5570 (5)	0.1046 (3)	0.5705 (3)	0.0435 (11)	
H5A	0.4822	0.1053	0.5749	0.052*	
C6	0.5806 (4)	0.1092 (2)	0.4952 (2)	0.0295 (8)	
C7	0.3761 (4)	0.1172 (3)	0.4054 (3)	0.0379 (10)	
H7A	0.3329	0.1153	0.4428	0.045*	
C8	0.3336 (4)	0.1219 (3)	0.3234 (3)	0.0389 (10)	
H8A	0.2572	0.1237	0.2943	0.047*	
C9	0.4292 (3)	0.1233 (2)	0.2927 (2)	0.0301 (8)	
H9A	0.4269	0.1262	0.2381	0.036*	
C10	0.7152 (3)	0.2476 (2)	0.3880 (2)	0.0280 (8)	
C11	0.6328 (4)	0.3117 (2)	0.3775 (2)	0.0296 (8)	
H11A	0.5559	0.2964	0.3638	0.036*	
C12	0.6627 (4)	0.3982 (3)	0.3869 (3)	0.0387 (10)	
H12A	0.6058	0.4399	0.3784	0.046*	
C13	0.7757 (5)	0.4221 (3)	0.4087 (3)	0.0512 (13)	
H13A	0.7950	0.4800	0.4149	0.061*	
C14	0.8604 (5)	0.3609 (3)	0.4213 (4)	0.0572 (14)	
H14A	0.9371	0.3767	0.4369	0.069*	
C15	0.8290 (4)	0.2753 (3)	0.4102 (3)	0.0383 (10)	
C16	1.0249 (4)	0.2038 (3)	0.4437 (4)	0.0682 (18)	
H16A	1.0740	0.2498	0.4615	0.082*	
C17	1.0564 (4)	0.1195 (3)	0.4393 (4)	0.0653 (17)	
H17A	1.1302	0.0973	0.4536	0.078*	
C18	0.9556 (4)	0.0745 (3)	0.4092 (3)	0.0451 (11)	
H18A	0.9506	0.0156	0.3996	0.054*	
C19	0.6751 (3)	0.1585 (2)	0.1718 (2)	0.0290 (8)	
H19A	0.6762	0.2183	0.1736	0.035*	
C20	0.6659 (4)	0.1168 (3)	0.0978 (2)	0.0353 (9)	
H20A	0.6611	0.1492	0.0515	0.042*	
C21	0.6641 (4)	0.0293 (3)	0.0932 (2)	0.0369 (9)	
H21A	0.6585	0.0019	0.0441	0.044*	
C22	0.6709 (3)	−0.0195 (2)	0.1641 (2)	0.0292 (8)	
C23	0.6710 (4)	−0.1098 (3)	0.1724 (3)	0.0398 (10)	
H23A	0.6657	−0.1451	0.1278	0.048*	
C24	0.6790 (4)	−0.1446 (3)	0.2465 (3)	0.0420 (11)	
H24A	0.6796	−0.2041	0.2526	0.050*	
C25	0.6864 (4)	−0.0915 (2)	0.3141 (2)	0.0303 (8)	
H25A	0.6913	−0.1167	0.3640	0.036*	
C26	0.6794 (3)	0.0278 (2)	0.2341 (2)	0.0231 (7)	
P1	0.33055 (9)	0.18918 (6)	0.06223 (6)	0.0258 (2)	
F1	0.42475 (18)	0.23551 (13)	0.13286 (12)	0.0291 (5)	
F2	0.2498 (2)	0.17991 (17)	0.12174 (15)	0.0450 (6)	
F3	0.4115 (2)	0.20007 (16)	0.00312 (14)	0.0392 (6)	
F4	0.3873 (2)	0.09807 (14)	0.09090 (15)	0.0417 (6)	
F5	0.2744 (2)	0.28140 (14)	0.03324 (14)	0.0372 (5)	
F6	0.2350 (2)	0.14372 (16)	−0.00851 (15)	0.0435 (6)	
Cl1	−0.0040 (4)	0.7352 (5)	0.2789 (3)	0.279 (3)	
Cl2	0.0137 (4)	0.5589 (5)	0.3252 (4)	0.302 (3)	
C27	0.0177 (11)	0.6755 (13)	0.3551 (10)	0.214 (8)	
H27A	−0.0402	0.6863	0.3836	0.256*	
H27B	0.0918	0.6888	0.3915	0.256*	

### Atomic displacement parameters (Å^2^)

**Table:**

	*U*^11^	*U*^22^	*U*^33^	*U*^12^	*U*^13^	*U*^23^
Ir1	0.02483 (8)	0.01435 (7)	0.02045 (8)	−0.00010 (5)	0.00159 (5)	−0.00110 (5)
N1	0.0284 (16)	0.0174 (14)	0.0222 (15)	0.0003 (12)	0.0064 (12)	0.0015 (11)
N2	0.0368 (18)	0.0195 (15)	0.0300 (17)	0.0019 (13)	0.0133 (14)	0.0004 (12)
N3	0.0291 (17)	0.0183 (15)	0.046 (2)	−0.0023 (13)	0.0033 (15)	−0.0015 (14)
N4	0.0296 (19)	0.0245 (17)	0.070 (3)	−0.0053 (15)	−0.0045 (18)	−0.0040 (17)
N5	0.0264 (16)	0.0185 (14)	0.0221 (15)	−0.0024 (12)	0.0025 (12)	−0.0014 (11)
N6	0.0253 (15)	0.0186 (14)	0.0250 (15)	0.0012 (12)	0.0071 (12)	0.0016 (11)
C1	0.038 (2)	0.0158 (16)	0.0241 (18)	0.0026 (15)	0.0014 (16)	−0.0021 (13)
C2	0.059 (3)	0.028 (2)	0.030 (2)	0.0075 (19)	−0.007 (2)	−0.0012 (16)
C3	0.102 (5)	0.031 (2)	0.021 (2)	0.012 (3)	−0.004 (2)	0.0003 (17)
C4	0.110 (5)	0.028 (2)	0.026 (2)	0.012 (3)	0.021 (3)	0.0009 (17)
C5	0.078 (3)	0.027 (2)	0.033 (2)	0.008 (2)	0.026 (2)	0.0020 (17)
C6	0.049 (2)	0.0155 (17)	0.0236 (18)	0.0025 (16)	0.0089 (17)	−0.0006 (13)
C7	0.033 (2)	0.030 (2)	0.056 (3)	0.0010 (17)	0.021 (2)	0.0007 (18)
C8	0.027 (2)	0.029 (2)	0.058 (3)	0.0014 (17)	0.0057 (19)	0.0043 (19)
C9	0.029 (2)	0.0230 (18)	0.034 (2)	0.0001 (15)	0.0000 (16)	0.0030 (15)
C10	0.041 (2)	0.0170 (17)	0.0242 (18)	0.0010 (15)	0.0048 (16)	−0.0019 (13)
C11	0.043 (2)	0.0205 (18)	0.0253 (19)	0.0011 (16)	0.0093 (17)	−0.0020 (14)
C12	0.058 (3)	0.0200 (19)	0.038 (2)	0.0073 (18)	0.013 (2)	−0.0022 (16)
C13	0.062 (3)	0.018 (2)	0.071 (3)	−0.005 (2)	0.012 (3)	−0.007 (2)
C14	0.051 (3)	0.029 (2)	0.085 (4)	−0.013 (2)	0.008 (3)	−0.009 (2)
C15	0.035 (2)	0.0201 (19)	0.052 (3)	−0.0029 (17)	−0.002 (2)	−0.0036 (17)
C16	0.031 (3)	0.039 (3)	0.119 (5)	−0.008 (2)	−0.008 (3)	−0.006 (3)
C17	0.027 (2)	0.045 (3)	0.111 (5)	0.004 (2)	−0.005 (3)	−0.002 (3)
C18	0.033 (2)	0.028 (2)	0.068 (3)	0.0030 (18)	0.003 (2)	−0.001 (2)
C19	0.032 (2)	0.0225 (18)	0.033 (2)	0.0021 (15)	0.0094 (17)	0.0082 (15)
C20	0.044 (2)	0.038 (2)	0.0251 (19)	0.0044 (19)	0.0111 (18)	0.0101 (16)
C21	0.050 (3)	0.037 (2)	0.0234 (19)	−0.0016 (19)	0.0101 (18)	−0.0004 (16)
C22	0.036 (2)	0.0276 (19)	0.0232 (18)	−0.0051 (16)	0.0061 (16)	−0.0022 (15)
C23	0.066 (3)	0.028 (2)	0.028 (2)	−0.008 (2)	0.015 (2)	−0.0095 (16)
C24	0.074 (3)	0.0171 (18)	0.036 (2)	−0.008 (2)	0.018 (2)	−0.0052 (16)
C25	0.045 (2)	0.0189 (17)	0.0273 (19)	−0.0042 (16)	0.0102 (18)	0.0030 (14)
C26	0.0269 (19)	0.0199 (17)	0.0215 (17)	−0.0008 (14)	0.0046 (14)	0.0018 (13)
P1	0.0312 (5)	0.0184 (4)	0.0244 (5)	0.0010 (4)	0.0015 (4)	−0.0036 (3)
F1	0.0335 (12)	0.0243 (11)	0.0242 (11)	−0.0019 (9)	−0.0015 (9)	−0.0027 (8)
F2	0.0416 (14)	0.0511 (16)	0.0445 (15)	−0.0082 (12)	0.0155 (12)	−0.0054 (12)
F3	0.0461 (15)	0.0417 (14)	0.0311 (12)	0.0004 (11)	0.0122 (11)	−0.0046 (10)
F4	0.0535 (16)	0.0168 (11)	0.0486 (15)	0.0020 (10)	0.0023 (12)	0.0010 (10)
F5	0.0427 (14)	0.0249 (11)	0.0362 (13)	0.0089 (10)	−0.0035 (11)	−0.0021 (9)
F6	0.0433 (14)	0.0369 (13)	0.0396 (14)	−0.0039 (11)	−0.0077 (11)	−0.0148 (11)
Cl1	0.177 (4)	0.470 (9)	0.169 (4)	−0.139 (5)	0.008 (3)	0.032 (5)
Cl2	0.106 (3)	0.407 (9)	0.381 (8)	−0.022 (4)	0.043 (4)	0.061 (7)
C27	0.116 (10)	0.34 (2)	0.197 (15)	0.054 (13)	0.065 (10)	0.096 (17)

### Geometric parameters (Å, º)

**Table:**

Ir1—C10	1.999 (4)	C11—H11A	0.9300
Ir1—N3	2.010 (3)	C12—C13	1.373 (7)
Ir1—N1	2.015 (3)	C12—H12A	0.9300
Ir1—C1	2.016 (4)	C13—C14	1.374 (7)
Ir1—N6	2.183 (3)	C13—H13A	0.9300
Ir1—N5	2.232 (3)	C14—C15	1.384 (6)
N1—C9	1.333 (5)	C14—H14A	0.9300
N1—N2	1.361 (4)	C16—C17	1.372 (7)
N2—C7	1.344 (5)	C16—H16A	0.9300
N2—C6	1.424 (5)	C17—C18	1.385 (6)
N3—C18	1.333 (5)	C17—H17A	0.9300
N3—N4	1.364 (4)	C18—H18A	0.9300
N4—C16	1.336 (6)	C19—C20	1.404 (6)
N4—C15	1.428 (5)	C19—H19A	0.9300
N5—C25	1.317 (5)	C20—C21	1.363 (6)
N5—C26	1.366 (4)	C20—H20A	0.9300
N6—C19	1.319 (5)	C21—C22	1.418 (5)
N6—C26	1.355 (4)	C21—H21A	0.9300
C1—C2	1.394 (6)	C22—C26	1.390 (5)
C1—C6	1.397 (6)	C22—C23	1.411 (5)
C2—C3	1.396 (7)	C23—C24	1.363 (6)
C2—H2A	0.9300	C23—H23A	0.9300
C3—C4	1.372 (8)	C24—C25	1.407 (5)
C3—H3A	0.9300	C24—H24A	0.9300
C4—C5	1.378 (7)	C25—H25A	0.9300
C4—H4A	0.9300	P1—F4	1.595 (2)
C5—C6	1.396 (6)	P1—F3	1.595 (3)
C5—H5A	0.9300	P1—F2	1.597 (3)
C7—C8	1.366 (7)	P1—F1	1.600 (2)
C7—H7A	0.9300	P1—F6	1.604 (2)
C8—C9	1.394 (6)	P1—F5	1.609 (2)
C8—H8A	0.9300	Cl1—C27	1.567 (14)
C9—H9A	0.9300	Cl2—C27	1.882 (19)
C10—C11	1.388 (5)	C27—H27A	0.9700
C10—C15	1.398 (6)	C27—H27B	0.9700
C11—C12	1.391 (5)		
			
C10—Ir1—N3	80.52 (14)	C13—C12—C11	120.2 (4)
C10—Ir1—N1	96.21 (14)	C13—C12—H12A	119.9
N3—Ir1—N1	173.28 (13)	C11—C12—H12A	119.9
C10—Ir1—C1	87.88 (14)	C12—C13—C14	120.4 (4)
N3—Ir1—C1	93.67 (15)	C12—C13—H13A	119.8
N1—Ir1—C1	80.29 (14)	C14—C13—H13A	119.8
C10—Ir1—N6	101.05 (13)	C13—C14—C15	118.5 (5)
N3—Ir1—N6	92.10 (13)	C13—C14—H14A	120.7
N1—Ir1—N6	94.29 (11)	C15—C14—H14A	120.7
C1—Ir1—N6	170.06 (13)	C14—C15—C10	123.3 (4)
C10—Ir1—N5	161.07 (13)	C14—C15—N4	122.4 (4)
N3—Ir1—N5	94.28 (12)	C10—C15—N4	114.2 (3)
N1—Ir1—N5	90.63 (11)	N4—C16—C17	107.7 (4)
C1—Ir1—N5	110.71 (12)	N4—C16—H16A	126.1
N6—Ir1—N5	60.74 (10)	C17—C16—H16A	126.1
C9—N1—N2	106.6 (3)	C16—C17—C18	105.7 (4)
C9—N1—Ir1	138.3 (3)	C16—C17—H17A	127.1
N2—N1—Ir1	115.0 (2)	C18—C17—H17A	127.1
C7—N2—N1	109.7 (3)	N3—C18—C17	110.1 (4)
C7—N2—C6	134.6 (4)	N3—C18—H18A	125.0
N1—N2—C6	115.7 (3)	C17—C18—H18A	125.0
C18—N3—N4	106.1 (3)	N6—C19—C20	121.4 (3)
C18—N3—Ir1	138.4 (3)	N6—C19—H19A	119.3
N4—N3—Ir1	114.9 (2)	C20—C19—H19A	119.3
C16—N4—N3	110.3 (4)	C21—C20—C19	120.7 (4)
C16—N4—C15	134.2 (4)	C21—C20—H20A	119.7
N3—N4—C15	115.5 (3)	C19—C20—H20A	119.7
C25—N5—C26	117.6 (3)	C20—C21—C22	119.2 (4)
C25—N5—Ir1	149.1 (3)	C20—C21—H21A	120.4
C26—N5—Ir1	93.3 (2)	C22—C21—H21A	120.4
C19—N6—C26	117.9 (3)	C26—C22—C23	116.2 (3)
C19—N6—Ir1	146.3 (3)	C26—C22—C21	115.6 (3)
C26—N6—Ir1	95.8 (2)	C23—C22—C21	128.1 (4)
C2—C1—C6	115.7 (4)	C24—C23—C22	119.1 (4)
C2—C1—Ir1	130.2 (3)	C24—C23—H23A	120.4
C6—C1—Ir1	114.1 (3)	C22—C23—H23A	120.4
C1—C2—C3	121.1 (5)	C23—C24—C25	120.6 (4)
C1—C2—H2A	119.5	C23—C24—H24A	119.7
C3—C2—H2A	119.5	C25—C24—H24A	119.7
C4—C3—C2	121.1 (5)	N5—C25—C24	121.8 (4)
C4—C3—H3A	119.5	N5—C25—H25A	119.1
C2—C3—H3A	119.5	C24—C25—H25A	119.1
C3—C4—C5	120.2 (4)	N6—C26—N5	110.2 (3)
C3—C4—H4A	119.9	N6—C26—C22	125.1 (3)
C5—C4—H4A	119.9	N5—C26—C22	124.6 (3)
C4—C5—C6	117.8 (5)	F4—P1—F3	90.16 (14)
C4—C5—H5A	121.1	F4—P1—F2	90.57 (15)
C6—C5—H5A	121.1	F3—P1—F2	179.07 (15)
C5—C6—C1	124.1 (4)	F4—P1—F1	90.17 (12)
C5—C6—N2	121.1 (4)	F3—P1—F1	89.89 (13)
C1—C6—N2	114.8 (3)	F2—P1—F1	89.53 (13)
N2—C7—C8	108.4 (4)	F4—P1—F6	90.47 (13)
N2—C7—H7A	125.8	F3—P1—F6	90.46 (14)
C8—C7—H7A	125.8	F2—P1—F6	90.11 (14)
C7—C8—C9	105.4 (4)	F1—P1—F6	179.27 (14)
C7—C8—H8A	127.3	F4—P1—F5	179.49 (15)
C9—C8—H8A	127.3	F3—P1—F5	89.43 (14)
N1—C9—C8	109.9 (4)	F2—P1—F5	89.85 (14)
N1—C9—H9A	125.1	F1—P1—F5	89.54 (12)
C8—C9—H9A	125.1	F6—P1—F5	89.83 (13)
C11—C10—C15	116.0 (3)	Cl1—C27—Cl2	110.9 (11)
C11—C10—Ir1	129.2 (3)	Cl1—C27—H27A	109.5
C15—C10—Ir1	114.7 (3)	Cl2—C27—H27A	109.5
C10—C11—C12	121.5 (4)	Cl1—C27—H27B	109.5
C10—C11—H11A	119.2	Cl2—C27—H27B	109.5
C12—C11—H11A	119.2	H27A—C27—H27B	108.1
			
C9—N1—N2—C7	−0.1 (4)	Ir1—C10—C15—C14	175.5 (4)
Ir1—N1—N2—C7	178.7 (2)	C11—C10—C15—N4	−178.0 (4)
C9—N1—N2—C6	178.2 (3)	Ir1—C10—C15—N4	−2.2 (5)
Ir1—N1—N2—C6	−3.0 (4)	C16—N4—C15—C14	9.1 (9)
C18—N3—N4—C16	0.1 (6)	N3—N4—C15—C14	−172.8 (5)
Ir1—N3—N4—C16	173.1 (4)	C16—N4—C15—C10	−173.1 (6)
C18—N3—N4—C15	−178.5 (4)	N3—N4—C15—C10	5.0 (6)
Ir1—N3—N4—C15	−5.4 (5)	N3—N4—C16—C17	−0.2 (7)
C6—C1—C2—C3	0.2 (6)	C15—N4—C16—C17	177.9 (6)
Ir1—C1—C2—C3	177.0 (3)	N4—C16—C17—C18	0.3 (8)
C1—C2—C3—C4	−0.3 (7)	N4—N3—C18—C17	0.1 (6)
C2—C3—C4—C5	0.3 (7)	Ir1—N3—C18—C17	−170.4 (4)
C3—C4—C5—C6	−0.2 (6)	C16—C17—C18—N3	−0.3 (7)
C4—C5—C6—C1	0.1 (6)	C26—N6—C19—C20	−0.5 (6)
C4—C5—C6—N2	−179.4 (4)	Ir1—N6—C19—C20	−178.8 (3)
C2—C1—C6—C5	−0.1 (5)	N6—C19—C20—C21	0.0 (7)
Ir1—C1—C6—C5	−177.4 (3)	C19—C20—C21—C22	0.4 (7)
C2—C1—C6—N2	179.4 (3)	C20—C21—C22—C26	−0.2 (6)
Ir1—C1—C6—N2	2.2 (4)	C20—C21—C22—C23	−179.6 (5)
C7—N2—C6—C5	−2.1 (6)	C26—C22—C23—C24	0.1 (7)
N1—N2—C6—C5	−179.9 (3)	C21—C22—C23—C24	179.5 (5)
C7—N2—C6—C1	178.3 (4)	C22—C23—C24—C25	0.4 (7)
N1—N2—C6—C1	0.5 (4)	C26—N5—C25—C24	0.0 (6)
N1—N2—C7—C8	0.0 (4)	Ir1—N5—C25—C24	−179.4 (4)
C6—N2—C7—C8	−177.8 (4)	C23—C24—C25—N5	−0.5 (7)
N2—C7—C8—C9	0.0 (4)	C19—N6—C26—N5	−179.5 (3)
N2—N1—C9—C8	0.1 (4)	Ir1—N6—C26—N5	−0.5 (3)
Ir1—N1—C9—C8	−178.2 (3)	C19—N6—C26—C22	0.7 (6)
C7—C8—C9—N1	−0.1 (4)	Ir1—N6—C26—C22	179.7 (3)
C15—C10—C11—C12	1.4 (6)	C25—N5—C26—N6	−179.2 (3)
Ir1—C10—C11—C12	−173.6 (3)	Ir1—N5—C26—N6	0.5 (3)
C10—C11—C12—C13	−1.4 (6)	C25—N5—C26—C22	0.6 (6)
C11—C12—C13—C14	0.1 (8)	Ir1—N5—C26—C22	−179.7 (3)
C12—C13—C14—C15	1.1 (8)	C23—C22—C26—N6	179.1 (4)
C13—C14—C15—C10	−1.0 (8)	C21—C22—C26—N6	−0.3 (6)
C13—C14—C15—N4	176.6 (5)	C23—C22—C26—N5	−0.6 (6)
C11—C10—C15—C14	−0.3 (7)	C21—C22—C26—N5	179.9 (4)

### Hydrogen-bond geometry (Å, º)

**Table:**

*D*—H···*A*	*D*—H	H···*A*	*D*···*A*	*D*—H···*A*
C9—H9*A*···F1	0.93	2.47	3.239 (4)	140
C9—H9*A*···F4	0.93	2.48	3.386 (5)	164
C16—H16*A*···F5^i^	0.93	2.46	3.018 (5)	118
C16—H16*A*···F6^i^	0.93	2.51	3.418 (6)	167
C7—H7*A*···F5^ii^	0.93	2.46	3.201 (5)	136
C25—H25*A*···F5^iii^	0.93	2.32	3.215 (4)	160
C27—H27*A*···F2^iv^	0.97	2.52	3.370 (13)	146

Symmetry codes: (i) *x*+1, −*y*+1/2, *z*+1/2; (ii) *x*, −*y*+1/2, *z*+1/2; (iii) −*x*+1, *y*−1/2, −*z*+1/2; (iv) −*x*, *y*+1/2, −*z*+1/2.
